# Dynamic Viscosity of the NaF-KF-NdF_3_ Molten System

**DOI:** 10.3390/ma15144884

**Published:** 2022-07-13

**Authors:** Alexei Rudenko, Alexander Kataev, Olga Tkacheva

**Affiliations:** Institute of High Temperature Electrochemistry, Ural Branch of the Russian Academy of Sciences, 620067 Ekaterinburg, Russia; lrizonl@gmail.com (A.R.); aleksandr_kataev@mail.ru (A.K.)

**Keywords:** dynamic viscosity, rotational viscometry, molten fluorides, neodymium fluoride

## Abstract

The dynamic viscosity (η) of the molten system (NaF-KF)_eut_-NdF_3_ containing NdF_3_ in an amount from 0 to 15 mol.% was studied by rotational viscometry using a high-temperature rheometer, FRS 1600. Viscosity measurements were carried out in the temperature range from liquidus to 1153 K. The measurement procedure was tested on the (LiF-NaF-KF)_eut_ melt. The choice of the parameter shear rate was carried out according to the viscosity and flow curves. Viscosity does not depend on shear rate, and therefore the investigated melts behave like Newtonian fluids, in the range of 9–19 s^−1^. The experimentally obtained viscosity values for (NaF-KF)_eut_-NdF_3_ melts in a wide temperature range are described by an exponential equation. In the coordinates ln(η) = *f*(1/T), they are straight lines; however, their temperature coefficients are noticeably different, which indicates significant impacts of composition and temperature.

## 1. Introduction

Mixtures of molten fluoride salts, due to their thermophysical, chemical and hydrodynamic properties, are widely used in various technological processes: as solvents for the electrolytic production of many metals and alloys, as fuel salts and as coolants in molten salt nuclear reactors (MSR), in systems for concentrating solar energy (CSP) [1,2,3], etc. At present, interest in MSR has increased due to the need to utilize highly active actinides (Np, Am, Cm). A promising prospect is a transmutation of long-lived actinides in MSR-burners. In this case, the concentration of actinides in fluoride melt can reach 20% [4]. However, the development in this direction is hampered by the lack of information regarding the physicochemical properties of molten alkali fluoride mixtures containing actinide fluorides and fission products. The study of such systems is time-consuming and expensive; therefore, measurements are carried out using simulators. Neodymium fluoride (NdF_3_) can serve as a simulator of AmF_3_ and CmF_3_, since they have similar thermodynamic properties, crystal lattice parameters and physicochemical characteristics [5].

An important physicochemical property of molten salts, which is necessary for assessing mass, heat transfer and hydrodynamic processes in electrolytic cells and reactors, is viscosity.

There are several fundamentally different approaches to measuring the viscosity of molten salts. They usually include capillary, ball drop, vibration, oscillatory and rotary methods, which are suitable for various temperature and viscosity ranges [6,7,8,9,10]. Nevertheless, special requirements are imposed on the structural materials of measuring devices and the measurement procedure for molten mixtures of fluoride salts. The melts must not interact with either the container material or the ambient atmosphere. Therefore, the oscillatory [11,12,13,14,15,16] or rotational methods [17,18,19] are used, as a rule, to measure the viscosity of fluoride melts. The capillary and falling ball methods, developed for less aggressive salts, must be modified for fluorides. Thus, the authors [20] developed an X-ray falling-ball viscometer instead of an optical one.

The methods of torsional vibrations of pendulums suspended on an elastic thread have received the greatest attention for determining the viscosity of molten halides and their mixtures. They consist in measuring the period and amplitude of the attenuation. When working with oscillatory pendulum viscometers, it is necessary to accurately know the inertia moment of the suspension system. Its direct determination at high temperatures is a difficult task, since it is impossible to accurately reproduce the conditions under which the viscosity of the molten salt is measured. Therefore, relative measurements are carried out and the device is calibrated against melts with a known viscosity. Any changes in the suspension system of the pendulum are accompanied by repeated measurements of the device constant.

The vibration method for measuring viscosity is widely used to study molten metals and slags. The sensitivity of viscometers significantly depends on the shape and mass of the probe immersed in the melt. The probe material must be chemically resistant to melts in order to exclude corrosion and the associated changes in instrument parameters.

The rotational method is based on the measurement of the force acting on the rotor (torque) during its rotation at a constant angular velocity (rotational speed) in the melt. The liquid under study is placed in a small gap between two cylinders. One of the cylinders remains stationary throughout the measurement; the other, called the rotor, rotates at a constant speed. The rotational motion of the rotor is transmitted to another surface through the movement of a viscous medium. Thus, the torque of viscometer rotor is a measure of viscosity.

Measurement of the molten salts viscosity at high temperature is a complex experimental task, and confirmation of this is the serious disagreement between the data of different authors. The discrepancy between the experimental data is determined by the quality (purity) of the salts used, method of investigation and type of viscometer.

The viscosity of the molten (LiF-NaF-KF)_eut_ (or FLiNaK) has been obtained by many researchers, since it is considered a promising medium for use as a coolant in molten salt reactors [10,21,22]. The measurements were carried out, as a rule, by the oscillatory or rotational method. It should be noted that measurement results often differ, regardless of the method used. Tasidou et al. [22] critically reviewed all known experimental data on the viscosity of FLiNaK, guided by the following criteria: a wide range of values should be presented with an accuracy of at least 1%, and an estimate of the measurement error should be given. The data of Janz [23] were considered as the most reliable, which, in turn, were based on the results of Torklep and Oye [11]. They carried out very thorough measurements by the oscillation method using a procedure that does not require separate instrument calibration. The error of the method was estimated to be 1%. The primary dataset (according to [22]) also included results obtained by An et al. [17] via the rotational method using a Brookfield type viscometer. The results of [12,13,14], also marked as the most reliable, were obtained by the oscillation method. The kinematic viscosity of FLiNaK was measured, and the dynamic viscosity was calculated using the density equation. An error of 2.5% was reported. Tobias [19] used a Zahn-type viscometer cup and measured the FLiNaK viscosity with an accuracy of 5%. These data also fell into the primary dataset.

The “secondary” data on the FLiNaK viscosity include the results of works [15,16,19] obtained both by oscillatory and rotational methods. Cohen [18] performed three series of FLiNaK viscosity measurements: one with the modified Brookfield viscometer and two with a capillary viscometer. The measurement error was 10%. Merzlyakov [15] measured the kinematic viscosity of FLiNaK over a wide temperature range using the torsional vibration damping method. The error calculation was not presented. The viscosity values presented in [15,18] are significantly higher than those of [17]. Vriesema [24] measured the kinematic viscosity of the FLiNaK melt with an error of 2% using a Venturi device to measure fluid flow. These results correlate with the data of the secondary group at a high temperature, and are similar to those of the primary dataset in the temperature range closer to the eutectic melting point.

The purpose of this work was to measure the dynamic viscosity of the molten mixtures (NaF-KF)_eut_. containing NdF_3_ in an amount of 0–15 mol.% in the temperature range from liquidus to 1153 K. To measure the viscosity of fluoride melts, a high-temperature rotational rheometer (viscometer)was applied, which was designed to determine the viscosity of molten electrolytes in a wide temperature range (300–1500 °C). In order to prove the reliability of the rotational technique, it was tested on (LiF-NaF-KF)_eut_. with a well-known viscosity.

## 2. Materials and Methods

### 2.1. Preparation of Salt Mixtures

The eutectic mixtures (mol.%) 40NaF-60KF and 46.5LiF-11.5NaF-42KF were prepared with the following chemicals: lithium fluoride LiF (mass fraction of LiF 99.0%) (CJSC VECTON), sodium fluoride NaF (mass fraction of LiF 99.0%) (LLC “GRANCHIM”), potassium fluoride acidic KF‧HF (mass fraction of KF‧HF 99-101%) (LLC “GRANCHIM”).

The mixtures were prepared by direct fusion of the components in a glassy carbon crucible. KF·HF was used instead of hygroscopic KF. KF·HF decomposes at 673–773 K; the released HF prevents the hydrolysis of salts and simultaneously fluorinates oxygen-containing impurities. The mixture was kept at 923 K for at least 2 h. After cooling, the sample was transferred to a glovebox with an inert argon atmosphere. The preparation technique is described in detail elsewhere [25]. The contents of LiF, NaF and KF in the prepared sample were confirmed by the elemental chemical analysis (Li, Na, K) carried out using ICP-OES (iCAP 6300 Duo, Thermo Scientific, Waltham, MA, USA).

The neodymium trifluoride (NdF_3_) was prepared by hydrofluorination of Nd_2_O_3_ according to technique described in [25]. The XRD confirmed the presence of a single hexagonal phase of NdF_3_. Analysis of the oxygen content using the oxygen analyzer LECO OH836 indicated that its content in the prepared NdF_3_ does not exceed 0.04 wt%.

### 2.2. Liquidus Temperature of (NaF-KF)_eut_-NdF_3_

The NdF_3_ additions to the molten (NaF-KF)_eut_ led to a significant change in the liquidus temperature of the mixture. Therefore, the liquidus temperatures of the (NaF-KF)_eut_ compositions containing 0, 5, 7.5, 10 and 15 mol % NdF_3_ were measured by thermal analysis, which consisted in recording the temperature during cooling. The obtained values of the liquidus temperature are listed in Table 1.

The measurements were carried out in an inert (Ar) atmosphere. The preliminarily prepared composition was loaded into a glassy carbon crucible and placed in a quartz cell with a tight lid, in which holes were performed for a thermocouple (Pt/Pt(Rh)) and an inert gas inlet/outlet.

### 2.3. Viscosity Measurement Technique

**Figure 1:**
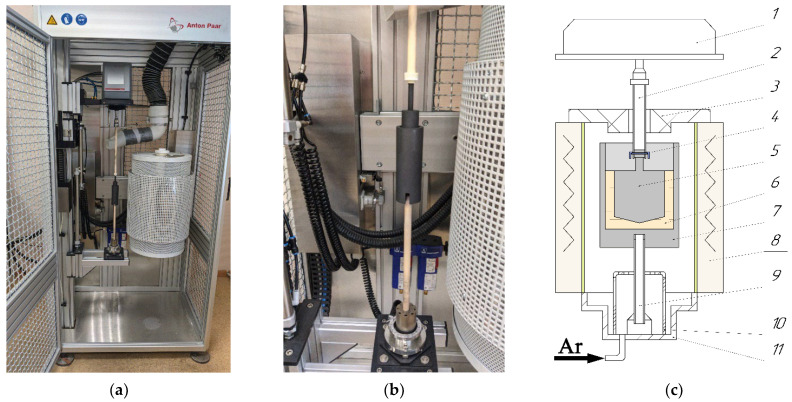
Exterior of rheometer FRS-1600 (**a**). Illustration of outer and inner cylinders (**b**) and scheme of the rheometer (**c**): 1—measuring head; 2—upper ceramic rod for fixing the rotor; 3—heat insulator cover; 4—system for attaching the rotor to the upper rod (pin + restrictive ring); 5—rotor (inner cylinder); 6—melt; 7—crucible (outer cylinder); 8—resistance shaft furnace with vertically arranged SiC heaters; 9—lower ceramic rod for mounting the crucible; 10—gas divider; 11—bottom cover with heat-insulating inserts.

The operating principle of the FRS-1600 rheometer (Anton Paar GmbH, Graz, Austria), is similar to that of the Brookfield viscometer [26]. The difference is that the outer cylinder is stationary, while the inner cylinder (rotor) rotates at a constant speed. The scheme of the rheometer and its illustration are presented in Figure 1. The investigated sample (melt) is placed between two graphite cylinders in a small gap (2 mm). The rotor is attached to the measuring “head” located at the top of the rheometer. The air-assisted pneumatic motor provides frictionless synchronous motion of the rotor, which increases the measurement sensitivity and allows measuring sufficiently low melt viscosity. A Carbolite STF16/180 electric resistance shaft furnace is used to heat and cool the sample.

The viscosity was measured in an inert gas atmosphere. The thermal expansion of the measuring system (change in the gap width) is automatically controlled by a rheometer.

## 3. Results and Discussion

### 3.1. Parameter “Shear Rate”

The basic law that underlies the rotational method and describes the flow of an ideal fluid is Newton’s law [27]:

(1) $$\tau =\;\eta \cdot \dot{\gamma },\;$$

where *τ* is the shear stress; *η* is the dynamic viscosity; $\dot{\gamma }$ is the shear rate.

To obtain the correct viscosity values, it is necessary that the laminar Newtonian flow is developed in the sample. This means that it takes time for the melt to start moving at a rate corresponding to the applied shear stress. The viscosity’s dependence on the shear rate was determined from the flow and viscosity curves.

**Figure 2:**
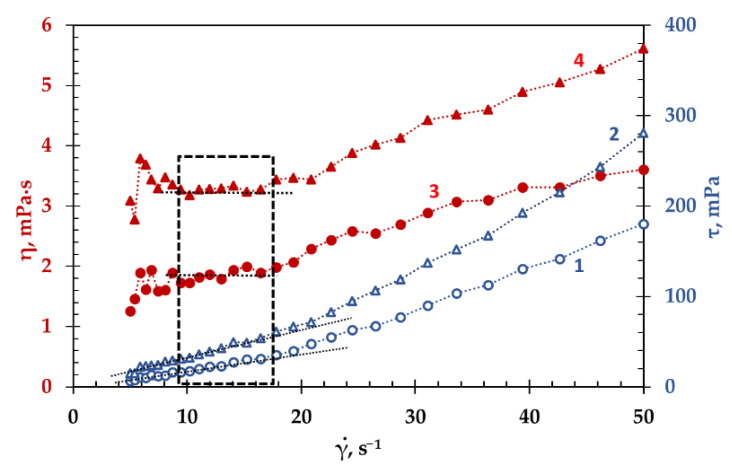
The flow (blue lines) and viscosity (red lines) curves of the (LiF-NaF-KF)_eut_ (1, 3) at 1023 K and (NaF-KF)_eut_-15NdF_3_ (2, 4) at 1273 K.

The flow curve depicting the relationship between the shear stress and the shear rate (*τ* = *f*($\dot{\gamma }$)), and viscosity curve, which represent the dependence of the viscosity of the test melt on shear rate (*η* = *f*($\dot{\gamma }$)), were obtained at constant temperature. The flow and viscosity curves of the (LiF-NaF-KF)_eut_ at 1023 K and (NaF-KF)_eut_-15NdF_3_ at 1273 K are presented in Figure 2. The flow curves are in blue, and the viscosity curves are in red in this figure.

**Figure 3:**
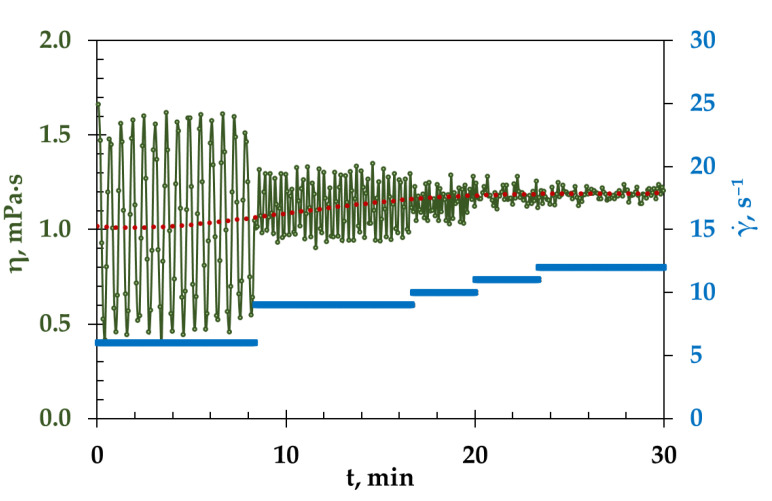
Viscosity of molten (LiF-NaF-KF)_eut_ at different shear rates (blue lines) with a fixed measurement time for each point (5 s).

The area of laminar flow can be determined both from the viscosity and flow curves. It follows from the Figure 2 that the values of the maximum shear rate at which the transition from laminar to turbulent flow occurred, determined from both curves, are in a good agreement. This transition occurs at a shear rate of 19 s^−1^. However, the viscosity curves indicate that a large spread of points was observed for both compositions at low shear rates (below 9 s^−1^). This was due to the applied measurement conditions, under which the specified measurement time did not correspond to the rate of the laminar flow. For example, Figure 3 illustrates the viscosity of the melt (LiF-NaF-KF)_eut_ at different shear rates, and the measurement time of each point was always constant: 5 s. The greatest scatter of points (marked in green in Figure 3) relative to the average value (red line) was observed at low shear rates. The higher the shear rate (under conditions of laminar flow), the smaller the spread of values. The viscosity values for the (LiF-NaF-KF)_eut_ are scuttered within 1% at the shear rate of 12 s^−1^.

Thus, to increase the measurement time for each point at low shear rates, it is possible to obtain values with the minimum deviation from the average value, but this will lead to a significant increase in the duration of experiment. According to the viscosity curves (Figure 2), it is possible to determine the range of the shear rate parameter, in which the viscosity does not depend on the shear rate—that is, when the melt behaves like Newtonian fluid. This interval was 9–19 s^−1^ for both melts, (LiF-NaF-KF)_eut_ and (NaF-KF)_eut_. The viscosity temperature dependence was measured at a cooling rate of 2 deg/min and a constant shear rate of 12 s^–1^.

### 3.2. Viscosity of Molten (LiF-NaF-KF)_eut_

**Figure 4:**
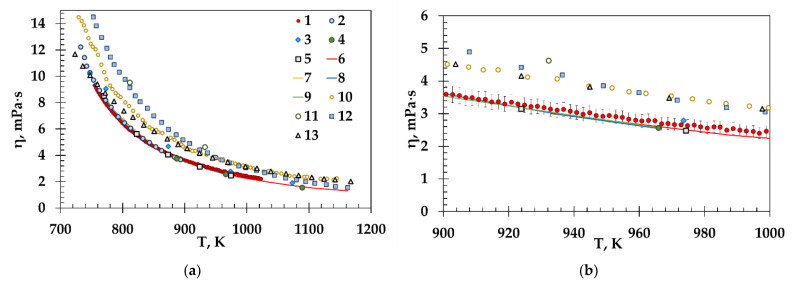
Viscosity of molten (LiF-NaF-KF)_eut_ in the temperature ranges 715–1170 K (**a**) and 900–1000 K (**b**) obtained by different studies: 1—this work, 2—An [17], 3—Cohen [18], 4—Tobias (capillary method) [19], 5—Torklep [11], 6—Cibulkova [13], 7—Kubikova [12], 8—Chrenkova [14], 9—Junz [23], 10—Merzlyakov [15], 11—Tobias (rotational method) [19], 12—Powers [28], 13—Vriesema [24]. Data notation in subfigure (**b**) is the same as in subfigure (**a**). Figure 4a was partially adapted with permission from Ref. [22], 2022, AIP Publishing LLC.

The rotational method was tested on the molten (LiF-NaF-KF)_eut_ with a well-known viscosity. The obtained viscosity, along with the literature data, is given in Figure 4a,b.

The obtained viscosities of the molten (LiF-NaF-KF)_eut_ are in a good agreement with the literature data from the first dataset (according to the reference correlation [22], as mentioned above) in a wide temperature range. In Figure 4b, drawn for the temperature range of 100 degrees, the scatter of the results is more clearly visible. Our data coincide with the data of works [11,12,13,14,17,19,28] within 7%. This confirms the reliability of the applied technique for measuring viscosity by the rotational method using the FRS-1600 rheometer.

### 3.3. Viscosity of Molten (NaF-KF)_eut_-NdF_3_

**Figure 5:**
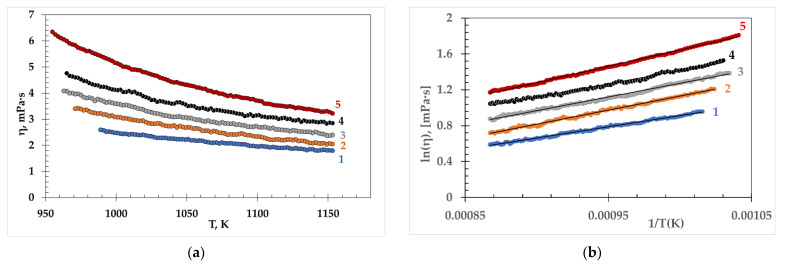
Viscosity of molten (NaF-KF)_eut_ containing NdF_3_ (mol.%): 1—0, 2—5, 3—7.5, 4 – 10, 5—1; measured (**a**) and calculated according to Equation (3) (**b**).

The viscosity of the (NaF-KF)_eut_-NdF_3_ melts with NdF_3_ content up to 15 mol.% in the temperature range from liquidus to 1153 K is presented in Figure 5a. The dependence is non-linear in a wide temperature range. The experimentally obtained values of the viscosity of fluoride melts in a wide temperature range are described by the Arrhenius equation:

(2) $$\eta \;=\;\eta_{0}\cdot \exp(\frac{E}{R\cdot T}),\;$$

where η is the dynamic viscosity, Pa‧s; $\eta_{0}$ is the pre-exponential factor defined from the experimental data, Pa; *E* is the activation energy for viscous flow, J/mol; *R* is the universal gas constant 8.3145 J/(K‧mol); *T* is temperature, K. In semilogarithmic coordinates, the viscosity temperature dependence is described by a general linear equation:

ln(η) = a + b/*T*, (3)

where a and b are the experimental constants. The viscosity temperature dependence of the (NaF-KF)_eut_-NdF_3_ melts in coordinates ln(η) = *f*(1/*T*) is shown in Figure 5b.

The equations for the temperature dependence of fluoride melts (NaF-KF)_eut_-NdF_3_ are given in Table 2.

**Figure 6:**
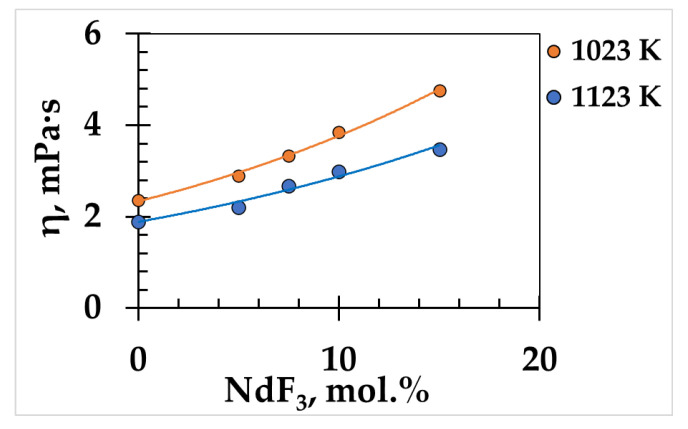
Effect of NdF_3_ additions on viscosity of (NaF-KF)_eut_-NdF_3_.

The viscosity of the (NaF-KF)_eut_-NdF_3_ melt grows with increasing NdF_3_ content (Figure 6).

Thus, the addition of 15 mol.% NdF_3_ led to an increase in the viscosity of the melt (NaF-KF)_eut_ by about two times.

The viscosity measurement error is the sum of the random error and the sum of the non-excluded systematic errors. The calculation of the non-excluded systematic error is caused by the error in measuring the temperature of the melt; viscosity (rheometer error); composition of the melt (by weighing the initial components of the melt and calculating the mass fractions of individual components); offset error—the difference between the measured and calculated viscosity values. The systematic component of the error consists of the error in measuring the melt temperature and the error in determining the composition of the melt (the error in weighing the initial components of the melt and calculating the mass fractions of individual components).

The FRS-1600 rheometer was calibrated according to the DGG1 standard (AntonPaar GmbH).

It was revealed that the relative error in measuring the viscosity of fluoride melts by the rotational method using the FRS-1600 rheometer does not exceed 1.5%. The error calculation is presented in Supplementary Materials.

### 3.4. Comparison of the Viscosity of (LiF-NaF-KF)_eut_ and (NaF-KF)_eut_

**Figure 7:**
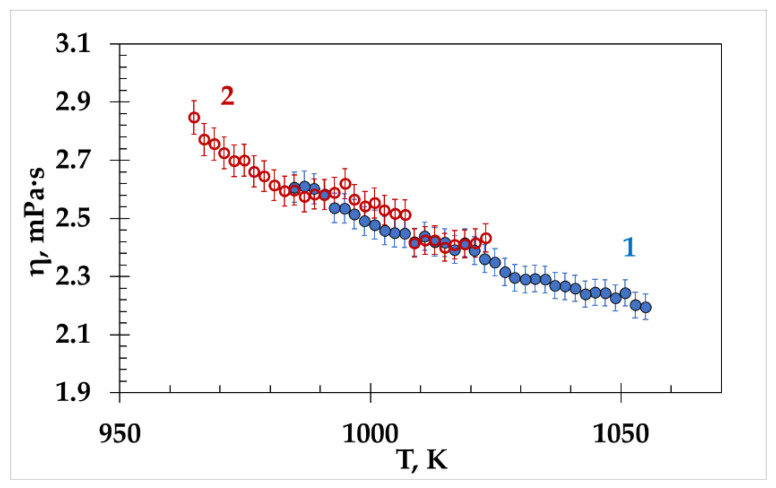
Viscosity of (NaF-KF)_eut_ (1) and (LiF-NaF-KF)_eut_ (2).

The viscosities of the molten mixtures (LiF-NaF-KF)_eut_ and (NaF-KF)_eut_ measured in the same temperature range are shown in Figure 7. The viscosity values of both eutectics coincide within 2%.

Therefore, the presence of the smallest Li^+^ cations in the ionic melt does not significantly affect the viscosity of the molten (LiF-NaF-KF)_eut_.

Viscous fluid flow is associated with the size, the configuration of particles and the nature of their interaction. To understand the phenomena of transport in ionic liquids, models of their structure are being developed. We tried to consider the obtained experimental results from the standpoint of the theory of the auto-complex model developed for the molten alkali halides and generalized in works [29,30]. According to this model, the ionic melts consist of single and complex ions; the latter arise in the melt due to the different polarizability of the electron shells of cations and anions (different ionic potentials—z/r (charge/radius)). Violation of symmetry in the mutual arrangement of ions leads to the fact that an ion–dipole interaction is superimposed on the ion–ion interaction. Grouping and coordination of more polarizable ions around less polarizable ones becomes energetically favorable. The distance between particles in the resulting associates or auto-complexes is almost the same as in crystals. As a result of the uneven distribution of the energy of thermal motion between particles, the formation of associates with different numbers of addends (from 1 to 6) is statistically probable. Calculations that take into account the ion–ion and ion–dipole interactions reveal that four-coordinated auto-complexes should predominate in alkali halide melts. With a further increase in their coordination number, the repulsive forces begin to prevail, as a result of which the binding energy decreases.

Therefore, the composition of single LiF and NaF melts can be represented as [(LiF_4_)^3−^ + 3Li^+^] and [(NaF_4_)^3−^ + 3Na^+^], and KF as [(FK_4_)^3+^ + 3F^−^]. In the melts of the latter fluoride, the formation of not complex anions, but complex cations, occurred, since the ionic potential of the cation (K^+^) is less than that of the fluorine anion (F^−^). For the CsF and RbF melts, the formation of complex cations is even more typical.

Estimation of the sum radii of complex anions, using the ionic radii (coordination IV) borrowed from [31], specified that their values are approximately the same for LiF and NaF—r((LiF_4_)^3−^) = 0.583 nm and r((NaF_4_)^3−^) = 0.623 nm—but differ for KF: r((FK_4_)^3+^) = 0.679 nm. The same trend was also observed for the values of the viscosity of these melts. The viscosities of LiF, NaF and KF, calculated using the experimental results of work [15] at 1300 K, were similar for LiF (1.40 mPa‧s) and NaF (1.38 mPa‧s) and differed from the viscosity of KF (0.97 mPa‧s).

In the binary or ternary mixtures, the complexing agent will be a cation with a high ionic potential. It coordinates the anions around itself, displacing the larger alkali cation into the second coordination sphere. As in the case of single salts, the formation of (MF_4_)^3−^ complexes with a coordination number of four will be preferable. Thus, the (NaF-KF)_eut_ melt will have the composition [(NaF_4_)^3−^ + 3K^+^], and the (LiF-NaF-KF)_eut_. Melt, −[(LiF_4_)^3−^ + Na^+^ + 2K^+^]. In both eutectics, the viscosity will be determined by the largest complex anions. Considering the principal contributions of (LiF_4_)^3−^ and (NaF_4_)^3−^ ions, with approximately the same size, to the melt viscosity, it is possible to assume one of the reasons for the same viscosity of (NaF-KF)_eut_ and (LiF-NaF-KF)_eut_ melts.

## 4. Conclusions

The procedure for determining the viscosity of molten fluoride mixtures using the FRS-1600 rotary rheometer was tested on molten (LiF-NaF-KF)_eut_ with a well-known viscosity over a wide temperature range. The shear rate parameter was determined, at which the viscosity does not depend on the shear rate, and melts (NaF-KF)_eut_ and (LiF-NaF-KF)_eut._ behave like Newtonian fluids.

The dynamic viscosity of the (NaF-KF)_eut_-NdF_3_ melts was measured at a constant shear rate of 12 s^−1^ in the temperature range from liquidus to 1153 K. The viscosity temperature dependence of the studied melts is described by the linear equation ln(η) = a + b/*T*. The viscosity of the (NaF-KF)_eut_-NdF_3_ melts rises with an increase in the NdF_3_ content: the addition of 15 mol.% NdF_3_ leads to an increase in the melt viscosity by about two times.

The viscosities of (LiF-NaF-KF)_eut_ and (NaF-KF)_eut_, measured in the same temperature range, coincide within 2%. It was assumed that one of the reasons for the same viscosity, which is generally determined by the size, the configuration of particles and their interaction, is the same configuration and similar sizes of the complex anions (LiF_4_)^3−^ and (NaF_4_)^3−^, predominantly present in the (LiF-NaF-KF)_eut_ and (NaF-KF)_eut_ melts, correspondently. Apparently, that the assumption requires further confirmation by experimental and simulation techniques.

## Figures and Tables

**Table 1:** Liquidus temperature of (NaF-KF)_eut_-NdF_3_ system.

NdF_3_ Content, mol.%	T, K
0	984
5	974
7.5	964
10	966
15	954

**Table 2:** Temperature dependence equations for (NaF-KF)_eut_-NdF_3_ melts.

Content NdF_3_, mol.%	ln(η) = a + b/T (Equation (3))	R^2^	T Range, K
0	ln(η) = −1.5898 + 2504.4/T	0.995	990–1153
5	ln(η) = −1.995 + 3126.4/T	0.996	980–1153
7.5	ln(η) = −1.777 + 3050.2/T	0.993	970–1153
10	ln(η) = −1.5147 + 2928.7/T	0.990	970–1153
15	ln(η) = −1.9912 + 3634.5/T	0.997	960–1153

## Data Availability

All data are freely available.

## Supplementary Materials

The following supporting information can be downloaded at: https://www.mdpi.com/article/10.3390/ma15144884/s1, Error calculation of viscosity measurement by rotational viscometry.
