# Supplementary material for: Dynamic Viscosity of the NaF-KF-NdF3 Molten System
Source: Materials (Basel). 2022 Jul 13;15(14):4884. doi: 10.3390/ma15144884 (PMC9324551; doi:10.3390/ma15144884)
Supplement: Supplementary file 1 [file materials-15-04884-s001.zip › materials-1783870-supplementary.pdf]

S1. Error calculation of viscosity measurement by rotational viscometry

S1.1. Method

Rotational viscometers use the standard principle of rotational viscometry: the measurement of viscosity is carried out by converting the torque required to rotate the instrument's rotor at a constant speed when immersed in the test molten sample. The liquid sample is placed in a small gap between two bodies. One of the bodies remains stationary throughout the measurement, the other (rotor) rotates at a constant speed. The rotational motion of the rotor is transmitted to another surface through the movement of a viscous medium. Thus, the torque of a rotational viscometer rotor is a measure of viscosity.

The basic law that underlies the rotational method and describes the flow of an ideal fluid is Newton's law:

$$\tau = \eta \cdot \dot{\gamma} \tag{S1}$$

where τ is the shear stress; η is the dynamic viscosity; $\dot{\gamma}$ is the shear rate.

S1.2. Equipment

To measure the viscosity of molten fluorides, a high-temperature rotational rheometer (viscometer) FRS-1600 (AntonPaar, Austria) was used, which is designed to determine the viscosity of molten electrolytes in a wide temperature range (600–1500°C). The FRS-1600 rheometer complies with the international standard ISO 7884-2 "Determination of viscosity using rotational viscometers".

S1.3. Sequence of operations

S1.3.1. Sample preparation

To prepare eutectics LiF-NaF-KF or NaF-KF, a weighed amount of salts were mixed in a glassy carbon crucible, which was placed in a furnace and heated to 923 K for 2 hours. After cooling the sample was transferred to a glove-box with an inert argon atmosphere. The absence of HF in the prepared salt was determined in several ways: using indicator paper, which was brought to the evaporating gas; or dissolve a salt sample in an aqueous solution and also determine the acidity.

Samples $(NaF\text{-}KF)_{eut}\text{-}NdF_3$ were prepared by melting a mixture of NaF-KF with certain additions of NdF_3 at a temperature of about 1000 K.

Before the experiment, the samples were crushed into pieces 5-8 mm in size, which makes it possible to place the sample more tightly in the crucible (outer cylinder). The crucible with sample, loaded into a special carrying container, transferred to the rheometer.

S1.3.2. Preparing the FRS-1600 rheometer for operation

Preparation of the FRS-1600 rheometer for operation is carried out in accordance with the work manual. Calibration of the FRS-1600 rheometer is carried out once during installation of the device or during scheduled maintenance using the DGG1 standard (Anton-Paar GmbH), which exhibits the properties of a Newtonian fluid in the temperature range (500-1400 °C). The certified DGG I viscosity at 1200 °C is 93.2 Pa·s, the measured FRS viscosity is 92.8 Pa·s. At 1200 °C, the average deviation of the viscosity of the measured sample does not exceed 0.5% relative to the viscosity values of the certified sample.

S1.3.3. Loading the test sample and measurements

After completion of all preparations, the test sample is loaded into the crucible, which is placed in the rheometer furnace and the measurement program is started.

S1.4. Estimation of the error in measuring the viscosity of melts

The measurement error is the sum of the random error and the sum of the non-excluded residuals of the systematic error.

Non-excluded systematic error is caused by:

- error in measuring the temperature of the melt;
- measurement error of the melt viscosity with a rheometer (rheometer error);
- the error in determining the composition of the melt (the error in weighing the initial components of the melt and calculating the mass fractions of individual components);
- an error in establishing the displacement - the difference between the measured and calculated viscosity values.

The systematic component of the error consists of the error in measuring the melt temperature, the error in determining the composition of the melt (the error in weighing the initial components of the melt and calculating the mass fractions of individual components), and the error in establishing the bias.

S1.4.1. Estimation of temperature measurement error

The temperature was measured with a Pt/Rh thermocouple (type S) attached to an FRS-1600 rheometer (the thermocouple is supplied with the rheometer). The error is:

$$\Delta t = 0.0025 \cdot t \quad \text{(S2)}$$

where t is the temperature, °C.

At melt temperature 880 °C (1153 K) Δ_t = 2.2 °C or δ_t = 0,25 %.

S1.4.2. Estimation of the error in measuring the viscosity of the melt with rheometer

The measurement error of the melt viscosity (the error of the FRS-1600 rheometer) was determined using the calibration data.

The FRS-1600 rheometer was calibrated according to the DGG1 standard (AntonPaar GmbH). The certified DGG I viscosity at 1473 K is 93.2 Pa·s, the measured FRS viscosity is 92.8 Pa·s. The measurement error of the melt viscosity with the rheometer FRS-1600 was estimated by the equation:

$$\delta_r = \frac{\Delta}{\eta_{CO}} \cdot 100\% = \frac{|\eta_{CO} - \eta_{meas}|}{\eta_{CO}} \cdot 100\% = \frac{93{,}2Pa \cdot s - 92{,}8Pa \cdot s}{93{,}2Pa \cdot s} \cdot 1 = 0{,}43\% \quad \text{(S3)}$$

S1.4.3. Estimation of the error in establishing the composition of the melt

The systematic error associated with establishing the composition of the studied melts is caused by errors in the weighing of the initial components. When preparing salts, each component (LiF, NaF, KF·HF) was weighed separately on a VLE-1023Cl electronic balance, the error of which is $\Delta_w = \pm 0.005$ g.

The mass fraction of the component in the melt φ_i is calculated by the formula:

$$\varphi_i = \frac{m_i}{M} \quad \text{(S4)}$$

where m_i – mass of sample of the i-th component, g;

M – total weight of melt sample, $m_{LiF} + m_{NaF} + m_{KF \cdot HF} = M$, g.

The expression for the error in establishing the mass fraction of the i-th component in the melt $\Delta\varphi_i$ is obtained by differentiating the original formula:

$$\Delta\varphi_i = \sqrt{\left(\frac{\partial f}{\partial m_i} \cdot \Delta m_i\right)^2 + \left(\frac{\partial f}{\partial M} \cdot \Delta M\right)^2} \quad \text{(S5)}$$

where Δm_i – error in establishing the mass of the i-th component of the melt, calculated by formula (S6), g;

ΔM – error in establishing the total mass of the sample, calculated by formula (S7), g;

$\frac{\partial f}{\partial m_i}$, $\frac{\partial f}{\partial M}$ – partial derivatives of the function φ_i (S4) with respect to the corresponding variable (the derivative is taken under the condition that at the time of taking all other variables are constants).

$$\Delta m_i = \sqrt{(\Delta m_{crucible})^2 + \left(\Delta m_{crucible + sample}\right)^2} = \sqrt{2 \cdot \Delta^2{}_b} = \Delta_b \cdot \sqrt{2} \quad (S6)$$

$$\Delta M = \sqrt{\left(\Delta_b \cdot \sqrt{2}\right)^2 + \left(\Delta_b \cdot \sqrt{2}\right)^2 + \left(\Delta_b \cdot \sqrt{2}\right)^2} = \sqrt{3 \cdot \left(\Delta_b \cdot \sqrt{2}\right)^2} = \Delta_b \cdot \sqrt{2} \cdot \sqrt{3} = \Delta_b \cdot \sqrt{6} \quad (S7)$$

$$\frac{\partial f}{\partial m} = \frac{1}{M}; \frac{1}{g} \quad (S8)$$

$$\frac{\partial f}{\partial m} = \frac{1}{M}; \frac{1}{g} \quad (S9)$$

The calculation results $\Delta\varphi_i$ are presented in Table S1.

Table S1. The error in establishing the mass fraction of the i-th component in the melt (40NaF-60KF)-NdF_3.

Component	ΔM, g	Δm, g	$\frac{\partial f}{\partial m}$, 1/g	$\frac{\partial f}{\partial M}$, 1/g	$\Delta\varphi$	δφ, %
NaF	0.01225	0.00707	0.002	-0.00017	0.00348	0.0108
KF·HF			0.002	-0.00031	0.00321	0.0035
NdF_3			0.002	-0.00080	0.00207	0.0300

The total error related to the accuracy of establishing the composition of the melt:

$$\delta\varphi_\Sigma = 1.1\sqrt{0.0108^2 + 0.0035^2 + 0.0300^2} = 0.035\ \% \quad (S10)$$

The accuracy of determining the composition of other melts under study can be taken as ≈ 0.035%, because the error in establishing the composition of this melt can be considered as maximum.

S1.4.4 Estimation of the error in establishing the displacement (difference between the measured and calculated viscosity values)

The error of establishing the displacement - the difference between the measured η and the calculated value of viscosity η_0 was considered using data on the obtained viscosity temperature dependence.

The temperature dependences of the NaF-KF melt obtained on the FRS-1600 rheometer are consistent with reliable literature sources, which is confirmed by the research results. Temperature dependencies, similarly established for other melts, may also be considered true.

The calculation results are given in Table S2.

Table S2. Calculation of the displacement at the maximum temperature of the experiment (T = 880 °C).

Melt composition	Уравнение	η_0. mPa·s	η. mPa·s	$\delta_c = \frac{\lvert\eta_i - \eta_0\rvert}{\eta_0} \cdot 100\%$

	температурной зависимости вязкости			
$40NaF\text{-}KF + 0\%\ NdF_3$	$\ln(\eta_0) = 3634.5/T – 1.99$	1.79	1.81	1.12
$40NaF\text{-}KF + 5\%\ NdF_3$	$\ln(\eta_0) = 2928.7/T – 1.51$	2.05	2.07	0.98
$40NaF\text{-}KF + 7.5\%\ NdF_3$	$\ln(\eta_0) = 3050.2/T – 1.78$	2.38	2.41	1.26
$40NaF\text{-}KF + 10\%\ NdF_3$	$\ln(\eta_0) = 3126.4/T – 1.99$	2.79	2.82	1.08
$40NaF\text{-}KF + 15\%\ NdF_3$	$\ln(\eta_0) = 2504.4/T – 1.59$	3.19	3.23	1.25
$(LiF\text{-}NaF\text{-}KF)_{uet}$ (750 °C)	$\ln \eta_0 = 4176.6/T – 3.20$	2.41	2.42	0.49

Remarks:
1) η_0 – viscosity values, established for the respective melts at the maximum temperature of the experiment;
2) η – viscosity values, measured using the rheometer for the respective melts at the maximum temperature

Due to the impossibility of estimating the random component of the measurement error, the measurement error of the melt viscosity is determined by the systematic component δ_η.

According to the rule of addition of systematic errors, the error in measuring the viscosity of melts is equal to:

$$\delta_\eta = 1.1\sqrt{(\delta_t)^2 + \left(\delta_{\varphi_\Sigma}\right)^2 + (\delta_c)^2 + (\delta_{rheo})^2} = 1.1\sqrt{(0.25)^2 + (0.035)^2 + (1.26)^2 + (0.43)^2} = 1.491 \approx 1.5\% \qquad \text{(S11)}$$

S2. Conclusions

The relative error in measuring the viscosity of melts $(LiF\text{-}NaF\text{-}KF)_{uet}$ and $(NaF\text{-}KF)_{eut} + NdF_3$ by rotational viscometry does not exceed 1.5 %.
